# Design of a Novel SiP Integrated RF Front-End Module Based on SOI Switch and SAW Filter

**DOI:** 10.3390/s24216994

**Published:** 2024-10-30

**Authors:** Xuanhe Wei, Youming Miao, Xiao Jin, Tian Hong Loh, Gui Liu

**Affiliations:** 1 College of Electrical and Electronic Engineering, Wenzhou University, Wenzhou 325035, China; 21451841026@stu.wzu.edu.cn (X.W.); 22461237086@stu.wzu.edu.cn (Y.M.); 23451248010@stu.wzu.edu.cn (X.J.); 2 Electromagnetic & Electrochemical Technologies Department, National Physical Laboratory, Teddington TW11 0LW, UK; tian.loh@npl.co.uk

**Keywords:** SiP integration, CA, diversity acceptance, RF front-end module

## Abstract

This paper proposes a novel System-in-Package (SiP) integrated architecture that incorporates Silicon-On-Insulator (SOI) switches and Surface Acoustic Wave (SAW) filters within the chip, aiming to fulfill the demands for miniaturization and multi-functionality for application in emerging wireless technologies. The proposed architecture not only reduces the integration complexity but also considers the architectural design of the integrated module, impedance matching techniques, and signal integrity for carrier aggregation (CA) technology realization. The feasibility of employing SOI switches and SAW filters based on SiP design has been validated through the trial production of a Sub-3GHz radio frequency (RF) front-end diversity receiver module. The resulting RF front-end module demonstrates exceptionally high packaging density and enhanced communication reliability, rendering it suitable for diverse applications in miniaturized RF systems.

## 1. Introduction

The radio frequency (RF) diversity receiver module plays a pivotal role in enhancing the reliability and anti-interference capabilities of wireless communication systems. The diversity combination technology at the receiving end can counter the multi-path fading effect, maximizing the system’s performance [1]. By employing various techniques, such as frequency and time diversity, multiple signals can be received on the same channel, effectively reducing the possibility of communication interruptions caused by environmental changes or signal attenuation. The application of this diversity technology is crucial in improving the stability and user experience of wireless communication systems. However, designing and implementing an RF diversity receiver module faces numerous challenges.

Firstly, with the advancement of emerging wireless communication technology, there is an ever-increasing demand for RF receivers to support more communication standards and frequency bands while achieving higher data transmission rates within smaller sizes. The microstrip matching circuit occupies a large area and is unsuitable for RF module design. Therefore, substrate capacitors and inductors have become the preferred choice in matching structure design. There are specific requirements for matching design, especially in multiband broadband matching. These requirements impose greater integration complexity on the design of RF diversity receiver modules without compromising performance. In response to the miniaturization trend in fifth-generation (5G) mobile communication equipment and the urgent demand for realizing high-density integration in 5G communication technology [2], RF module integration and packaging are progressing towards enhanced integration, lightweight design, and improved scalability. System-in-Package (SiP) packaging technology facilitates the integration of multiple chips into a single package, thereby minimizing the package size and increasing the package density [3,4]. Moreover, SiP packaging technology offers the potential for flexible design, allowing for the seamless combination of different chips and components based on their respective functions and requirements. This feature makes it particularly well suited for RF-related applications such as 5G millimeter-wave modules. This reduces the need for external connections while providing superior electromagnetic (EM) shielding effects and improving overall system performance and reliability. The advanced dual-side SiP module uses a double-sided Surface-Mounted Technology (SMT) and molding process to reduce its size and increase integration density effectively [5]. Reference [6] proposes a compact three-dimensional (3D) SiP design for a four-channel RF transceiver, demonstrating the stacking of silicon and aluminum nitride carriers interconnected through micro-bumps. Reference [7] shows a miniaturized RF front-end module, integrated with an antenna array, designed for ease of maintenance in 5G millimeter-wave communications. Furthermore, Reference [8] introduces a multi-input multi-output (MIMO) RF front-end module capable of operating in the 2 GHz to 5 GHz frequency band, incorporating an RF switch and low-noise amplifiers (LNAs) to enhance the performance.

Secondly, the demand for higher data rate transmission continues to grow alongside increasing integration density. Due to the high frequency of fourth-generation (4G) and 5G signals, there are transmission loss issues that necessitate improving the stability and reliability of the receiving system. Carrier aggregation (CA) technology has been widely employed in 4G and 5G networks to cater to the need for high-speed data transmission. CA technology significantly boosts bandwidth and data transmission capacity by combining multiple carrier channels, resulting in faster download and upload speeds for users. Furthermore, it optimizes network coverage and stability, improves spectral efficiency, and ultimately provides an enhanced user experience. The low-power RF receiver proposed in [9] supports multi-channel RF signals in CA scenarios, offering flexibility through various selection options based on module configuration and target applications. In [10], a CA-based five-way acoustic filter was proposed, optimizing the design of phase shifting networks to reduce the number of phase shifters and enhance the flexibility of signal channel multiplexing.

Thirdly, chip interconnection is pivotal in module design, essential for enabling more complex functionalities. RF receiving modules require integration with other system components, such as ADCs and AGCs. This necessitates that designers consider not only the performance of individual modules but also their interaction and compatibility. The flip chip technology is an advanced integrated circuit electronic packaging technique that enables higher input/output (I/O) density, shorter signal paths, improved heat dissipation, and enhanced reliability through direct attachment of the chip to the substrate. Reference [11] proposed an inverted chip interconnection structure based on gold bumps, suitable for realization of the single-chip microwave integrated circuit chips with the front side facing upwards.

Finally, with the progress of 5G mobile communication technology, there is a growing demand for improving the filter performance to meet the more challenging technical specifications. As a crucial component of the receiving module, the filter plays a decisive role in the overall received signal, influencing factors such as temperature drift and out-of-band suppression, which significantly impact the module’s performance. Selecting a suitable filter with low-temperature drift, effective out-of-band suppression, and minimal insertion loss poses a key challenge. Acoustic filter technology has witnessed significant advancements through novel architectures, innovative materials, and advanced modeling techniques, establishing itself as the predominant filter technology in contemporary mobile RF front-end applications [12,13]. A low-loss, high-isolation novel filter was designed and experimentally implemented, demonstrating the potential of this technology for effectively integrating band-in full-duplex systems in miniaturized RF front ends [14]. The incorporation of filters into RF front-end modules has become imperative. In [15], a chip was developed to combine SAW filters and a versatile power amplifier (PA) capable of operating at multiple frequencies and modes. A single-chip multiband RF front-end module integrating bulk acoustic wave filters, lamb acoustic wave filters, and RF SOI switches has been reported in [16].

In this study, we address the requirements for achieving highly dense integration in RF systems by selecting SOI switches and SAW filters as integrated components to design an SiP-based RF front-end diversity receiving module. Through an investigation of integration structures, RF transmission performance, and CA operating modes, we successfully integrate SOI switches with SAW filters on a single chip to achieve module miniaturization and high levels of integration. It offers extensive operational modes and diverse CA scenarios in Sub-3GHz frequency bands, exhibiting remarkable versatility and flexibility. Additionally, in contrast to conventional packaging forms, the RF front-end diversity reception module employed SiP packaging in this study, thereby enhancing the integration level. The implementation of the flip-chip design effectively optimized insertion loss. The SiP-based diversity receiving module offers advantages such as exceptional levels of integration and optimization capabilities for energy efficiency while accommodating heterogeneous material integrations. The design of the module was finally completed.

## 2. Integrated Architectures

**Figure 1:**
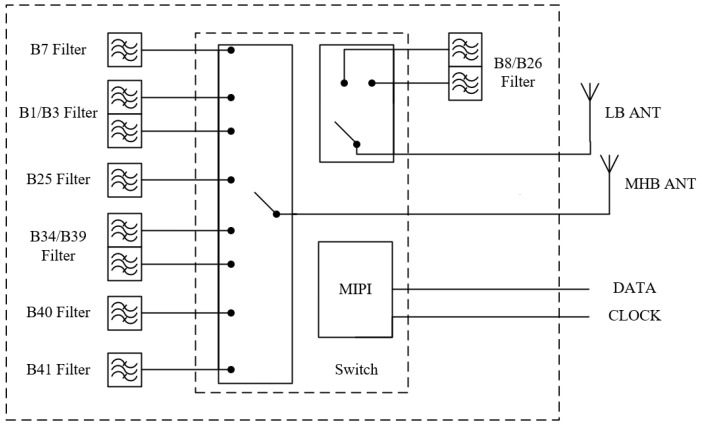
RF front-end diversity receiving module.

The operation of 5G mobile devices requires the use of a multitude of frequency bands for diverse communication functions, including the traditional GSM and 4G LTE bands. The RF front-end diversity reception module designed in this paper integrates switches and filters to optimize RF circuit performance, covering multiple frequency bands suitable for use in modern communication environments. Moreover, systematic enhancements and precision design optimizations were applied to both CA impedance and the RF link-matching network to fulfill the high data rate demands of the RF front-end system. The structural block diagram of the RF front-end diversity reception module is shown in Figure 1. The diversity reception module is equipped with two antennas, one for transmitting low-band (LB) signals and the other for transmitting middle-high-band (MHB) signals. The module includes seven filters, comprising three multiplex filters capable of simultaneously processing signals across two frequency bands. It features two external antennas for signal input. Additionally, Data and Clock signal lines are configured for MIPI (Mobile Industry Processor Interface) to control the switching of different channels. The module operates across multiple frequency bands, such as B1 and B3, as outlined in the frequency comparison table specific to Wideband Code Division Multiple Access (WCDMA) and other applicable standards. All the filters used correspond to the receiver (Rx) frequencies and are connected to the switches through board traces. By integrating MIPI as a switch controller and multiple acoustic filters on a substrate, multi-path selection and filter reuse are achieved, resulting in carrier aggregation. Table 1 details the operational frequency range and carrier aggregation (CA) scenarios for the RF diversity reception module. Specifically, “B0103” aggregates frequency bands B1 and B3, while “B3439” combines B34 and B39.

SOI technology offers significant advantages over Complementary Metal-Oxide-Semiconductor (CMOS) technology in low power consumption, high performance, high integration, and resistance to harsh environments. Partial depletion SOI MOSFETs are the mainstream technology in RF SOI systems [17]. The dielectric isolation characteristics of SOI materials enable full dielectric isolation without the latch effect, a small active area, a small parasitic capacitance, and a small leakage current in integrated circuits, making them ideal for use in harsh environments such as irradiation circuits and high temperatures. SAW filters are preferred for their capability to attain ultra-high-quality factors with extremely narrow bandwidth filtering functionality while still capable of maintaining low loss since they operate without internal direct current (DC). Additionally, the relatively simple manufacturing process allows for a higher degree of integration, which reduces the system complexity and overall cost.

The utilization of an RF switch for multi-path selection enables switching between non-CA and CA operating modes, encompassing a range of frequency bands from low-frequency B26 to high-frequency B7. In terms of link design, the feasibility of the CA operating mode is ensured through the optimization of the filter matching network. By adjusting the input impedance of the filter, one can ensure that the passband exhibits conductivity while maintaining a high impedance state for the remaining frequency bands. Proper adjustment of impedance matching enables coordinated performance between CA and non-CA operating scenarios, thereby optimizing the operational efficiency of the filter.

**Figure 2:**
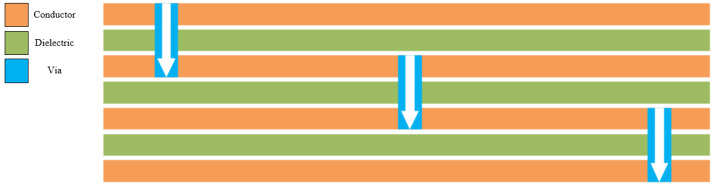
Chip substrate stacking setting.

In addition to various non-CA operating modes, there are ten CA operational modes, including B0103 performing carrier aggregation with B7 (B0103 CA B7), etc. Furthermore, the incorporation of SAW filters offers numerous advantages such as enhanced design flexibility, reduced insertion loss, improved reliability, exceptional EM interference resistance performance, a compact size, and a lightweight nature, as well as a cost-effectiveness that aligns with the requirements of RF systems. The filter’s matching structure, consisting of a wound inductor and surface-mounted capacitors, is positioned on the substrate. In terms of substrate design, a 4-layer metal substrate and a 3-layer dielectric-filled SiP package are employed. The specific configuration of the substrate lamination is illustrated in Figure 2. This design not only facilitates achieving the required substrate performance but also allows for independent selection based on specific needs, enabling optimization of both area and performance for integrating additional electronic components.

**Figure 3:**
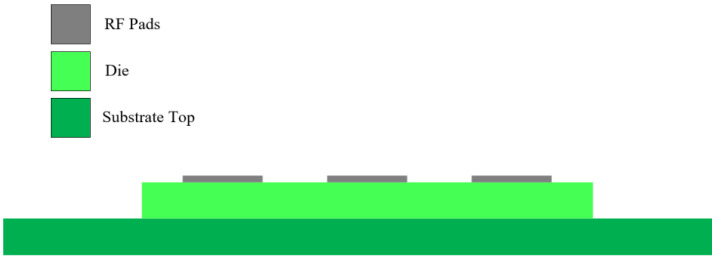
Die-stack configuration.

Integrating the chip with the substrate facilitates the incorporation of the matching structure into the filter and switch. Regarding interconnection methodology, this design employs flip-chip interconnection, wherein the chip is directly flipped over and connected to either the package housing or wiring substrate. The schematic diagram of the flip chip is illustrated in Figure 3. Compared to the lead bonding method, this technology significantly reduces signal transmission distance and overall footprint, thus facilitating packaging integration. Furthermore, due to the reduced transmission distance, the flip-chip welding connection mode ensures a more compact and stable configuration. Additionally, the flip-chip welding technique enables the selection of diverse interconnection structures based on different substrate materials, thus allowing for varied material compositions and structural arrangements within the final interconnection structure.

5G RF modules typically incorporate piezoelectric filters based on SAW and bulk acoustic wave (BAW) technologies, as well as switches utilizing SOI technology, which poses compatibility challenges with semiconductor processes like PA and LNA in the RF front-end module. To tackle this challenge, module design often adopts SiP mode to achieve integration and ensure scalability. This is accomplished by combining multiple bare chips and micro passive devices, manufactured using different integrated circuit processes, onto a compact substrate through advanced packaging techniques and a high-precision SMT process. In summary, to meet the miniaturization and scalability requirements of the RF front-end diversity receiver module, this paper utilizes SOI switches and SAW filters as module devices for realizing the SiP-based RF front-end diversity receiver module.

## 3. Carrier Aggregation and Impedance Matching

### 3.1. Carrier Aggregation

Carrier aggregation (CA) is a key technology for enhancing communication rates and capacities, providing a foundation for multi-band applications. It not only facilitates RF front-end integration but also addresses challenges in complex network environments. Moreover, it plays a crucial role in advancing new standards and technologies. In intricate network settings, signal quality and stability are compromised by interference and attenuation. By utilizing signals from multiple frequency bands and communicating through the optimal ones, CA technology improves signal quality and enhances communication reliability.

According to the Shannon–Hartley channel capacity formula, the channel capacity (maximum transmission rate) is determined by the transmission bandwidth *W*, output signal power *S*, and channel noise power *N*.

(1) $$C=Wlog(1+\frac{S}{N})$$

CA enables higher peak rates by amalgamating diverse spectrum blocks across both 5G new radio (NR) Frequency Range 1 (FR1) and Frequency Range 2 (FR2) bands. Within FR1 bands encompassing Frequency Division Duplex (FDD) and Time Division Duplex (TDD), various CA operation scenarios such as FDD plus FDD, FDD plus TDD, and TDD plus TDD can be employed to fulfill design requirements. That is, by increasing the *W* in the above equation, channel capacity can be increased.

**Figure 4:**
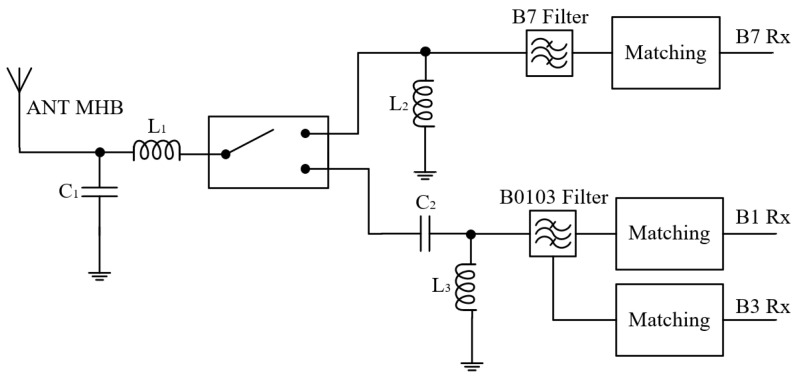
Schematic of B0103 CA B7 in the diversity receiving module. (ANT: antenna; Rx: receiver).

Figure 4 illustrates the circuit diagram of B0103 CA B7, showing the input and output ports designated as the antenna port and signal reception port, respectively. The antenna is connected to the switch via a matching network, which further connects to the filter. By simultaneously activating both branches of the switch that require aggregation, CA is achieved.

### 3.2. Matching Structure Adjustment and Parameter Optimization

To ensure the feasibility of the module, various scenarios simulating CA are conducted due to its working mode. By optimizing the design of its matching structure, it is imperative to guarantee excellent performance in non-CA operating mode and fulfill sufficient requirements in CA operating mode.

**Figure 5:**
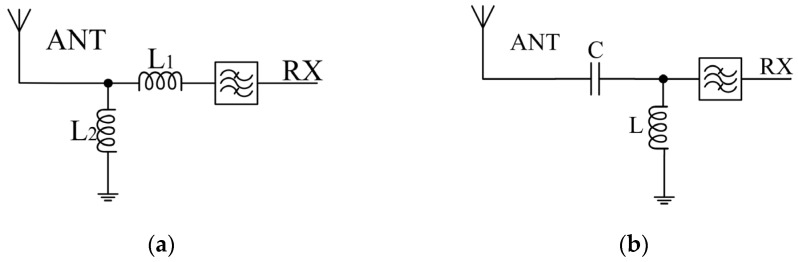
Schematic diagram of B0103 circuit under different matching structures: (**a**) L-matching; (**b**) LC-matching.

**Figure 6:**
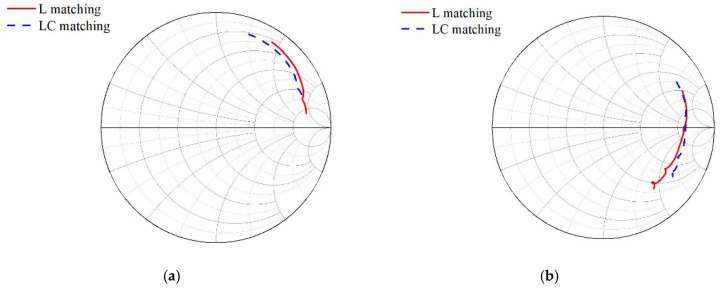
The variation of CA impedance under different matching structures in RF chip scenarios: (**a**) B0103 in B25 Band; (**b**) B0103 in B40/B41/B7 Bands.

Designing the matching circuit poses a challenge due to the requirement of supporting multiple frequency bands and impedances in different switch states. To address this, interconnect analysis using the Keysight Advanced Design System (ADS) 2021 software is performed on switch and filter models, enabling verification analysis of various matching structures to identify the most favorable structure for CA. The SOI switch and SAW filter are both products of Starshine Semiconductor Company. The model used in ADS is the S-parameter model of these products. Figure 5 illustrates the characteristics of the B0103 filter in the medium-to-high-frequency range, depicting two types of input impedance matching and filter connection methods. Here, the values of each variable are L1 = 0.4 nH, C = 4 pF, and L = L2 = 4 nH. These matching methods include inductive matching and inductive capacitive matching. The B0103 filter covers four distinct CA operating conditions. Figure 6 illustrates the simulated results of the corresponding CA impedance for each frequency band under two different input impedance-matching network configurations shown in Figure 5. Both configurations maintain the same RX matching to 50 ohms, namely inductance-matching (L-matching) and inductance–capacitance-matching (LC-matching).

In Figure 5, the common antenna impedance of the signal from the antenna port pair towards the matched filter is simulated to obtain the comparison results shown in Figure 6. Based on the simulation results in Figure 6, it can be observed that the two lines have opposite effects on the high- and low-frequency bands. The red line is more favorable for CA operations in the low-frequency range, while the blue line is more advantageous for CA operations at higher frequencies. The CA impedance will exert a substantial impact on the corresponding frequency band’s filter performance during CA operation. Moreover, different filters exhibit varying degrees of sensitivity or requirement towards the CA impedance. Therefore, a reasonable selection should be made based on design requirements.

**Figure 7:**
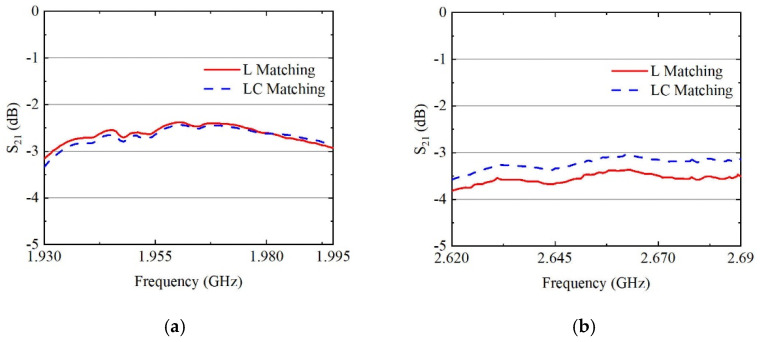
Insertion loss for the frequency range corresponding to the B0103 CA condition: (**a**) B25 band; (**b**) B7 band.

Based on the impedance data from Figure 6, we selected the two frequency bands with the most significant disparity for simulation analysis: B0103 CA B25 and B0103 CA B7. The results of the simulations are presented in Figure 7. Figure 7 shows that the high-frequency band B7 is significantly influenced by the two distinct matching methods, while the impact on the low-frequency band B25 is negligible. In comparison to LC-matching, L-matching exhibits almost no discernible effect on the overall performance of the B25 band; however, there is an average degradation of 0.4 dB in insertion loss at higher frequencies. This gap reveals that adjusting the CA impedance design serves as a crucial approach for achieving a performance trade-off within the diversity reception module. For different filters with varying input and output impedances, it is also necessary to optimize the matching structure of each filter using a similar method to achieve the best system matching.

## 4. Interconnect Structure

In the design of the RF chipboard, the antenna receives the desired working signal through switches and filters. However, due to the requirement for interconnections among the antenna, switch ports, and filters, the RF signal necessitates traversing various modules before reaching its final output pin. These connections are established through wiring, not as idealized in a schematic diagram. Therefore, it is crucial to simulate corresponding matching structures and the board to address the effects of these wiring connections on signal integrity.

**Figure 8:**
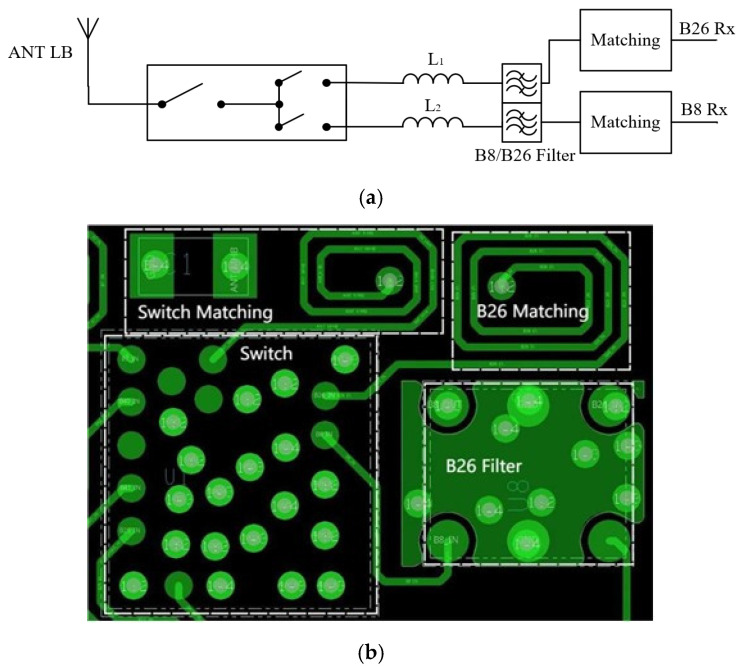
The schematic diagram and baseplate drawing of the B26 pathway: (**a**) schematic diagram; (**b**) baseplate drawing.

To illustrate the connection method and corresponding input matching of the B26 filter without CA, it is considered as an example depicted in Figure 8a, where a series inductor is utilized.

The designed module uses a winding inductor to complete the inductor configuration for impedance matching. The inductance of the trace is calculated based on the length *l* and width *W* of the trace. The inductance of the via is calculated based on the height *h* and diameter *d* of the via [18].

(2) $$L_{t}=2l\left(ln\frac{2l}{W}+0.5+0.2235\frac{W}{l}\right)$$

(3) $$L_{v}=\frac{h}{5}\left(1+ln\frac{4h}{d}\right)$$

Additionally, the calculation of winding inductance is more complex and requires different calculation formulas to be used depending on the situation. According to the calculation formulas for trace inductance and via inductance, the corresponding drawings are made based on the schematic diagram [19]. The inductance of a single-layer coil depends on the magnetic permeability *μ* of the material, dl_1_, and dl_2_ are line elements, and *R* is the distance between *dl*_1_ and *dl*_2_:

(4) $$L=\frac{\mu_{0}}{4\pi }\oint_{C_{1}}\;\oint_{C_{2}}\;\frac{dl_{1}\cdot {dl}_{2}}{R}$$

Based on this circuit diagram and calculation formula, we carry out a printed circuit board (PCB) design and implement the inductor as a winding inductor, as shown in Figure 8b. Figure 8b illustrates the switch, matching network, the B26 filter, and the B26 matching elements.

**Figure 9:**
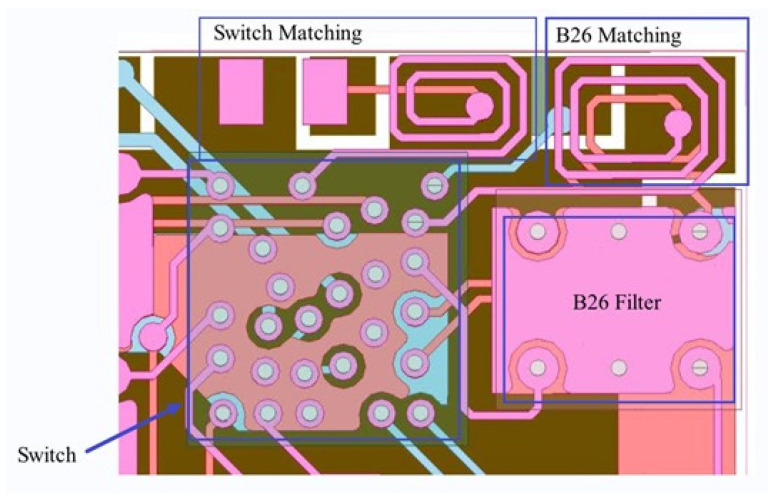
HFSS 3D EM simulation model diagram of the B26 pathway.

Equations (2)–(4) are used for approximate length estimates in substrate design but do not play a direct role in the simulations. To enhance the precision and efficiency of the design, as well as optimize design challenges, an Ansys High-Frequency Structure Simulator (HFSS) full-wave EM simulation model is established for the substrate, and EM simulations are conducted to assess the EM impacts on the circuit, thereby mitigating potential influences on semiconductor devices and other components. Drawing the base plate according to the schematic diagram enables simulation of its EM influence using the base plate file. The HFSS model generated from the board file can be seen in Figure 9.

**Figure 10:**
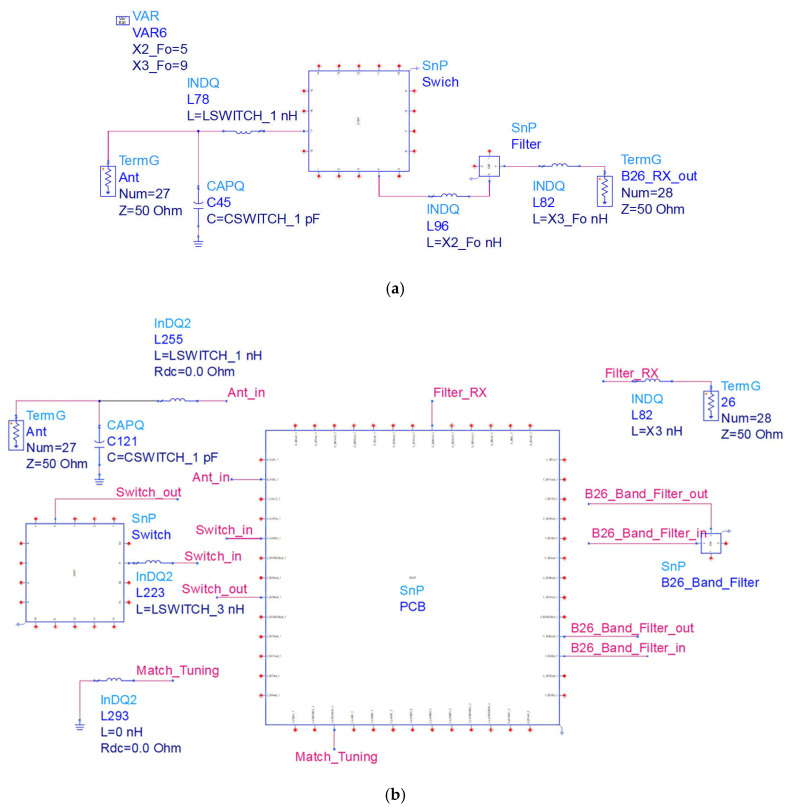
EM and schematic simulation models of B26: (**a**) schematic; (**b**) EM.

The HFSS full-wave EM simulation model is used for simulating the EM effects between traces or components, to validate and simulate real-life scenarios. The comparison results between the simulation results in the model and the circuit simulation results in the schematic diagram are shown in Figure 10.

The impact of EM effects can be observed by comparing the simulation results obtained from HFSS EM simulation with the circuit schematic simulation. Figure 10 presents a comparative analysis between the HFSS full-wave EM model simulation results and the ADS circuit schematic simulation results, illustrating both the insertion loss, *S*_21_, and the input impedance of the B26 filter link.

A schematic model and an EM model are built in ADS 2021 software. The EM model generates the track and parasite within the substrate model based on the ports, as shown in Figure 10b.

**Figure 11:**
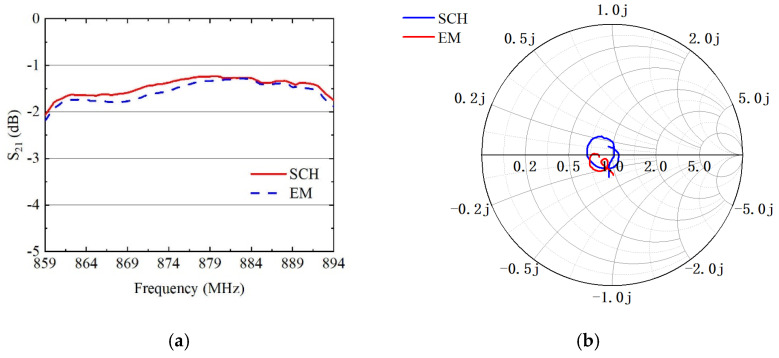
EM and schematic simulation results of B26: (**a**) insertion loss; (**b**) input impedance Smith diagram.

From Figure 11a, it can be seen that the EM simulation results are approximately 0.2 dB worse than the schematic simulation results. In Figure 11b, it can be seen that the input impedance of B26 is significantly more dispersed than the ideal case due to parasitic effects. The simulation results of EM and the schematic exhibit consistent impedance position and insertion loss, demonstrating overall agreement between the two methods.

**Figure 12:**
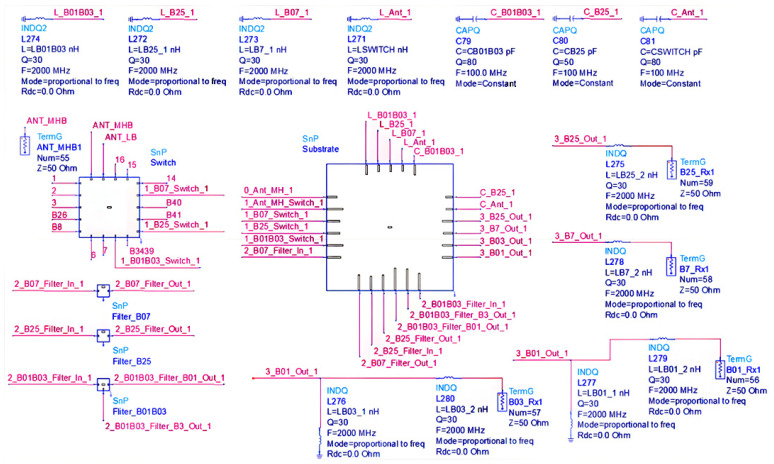
Schematic of B0103 CA B25 in ADS with EM simulation result.

**Figure 13:**
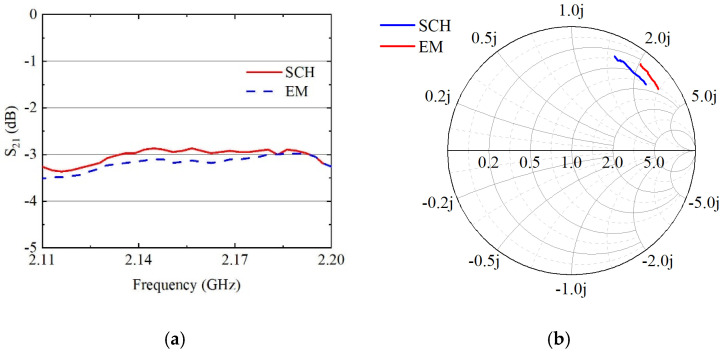

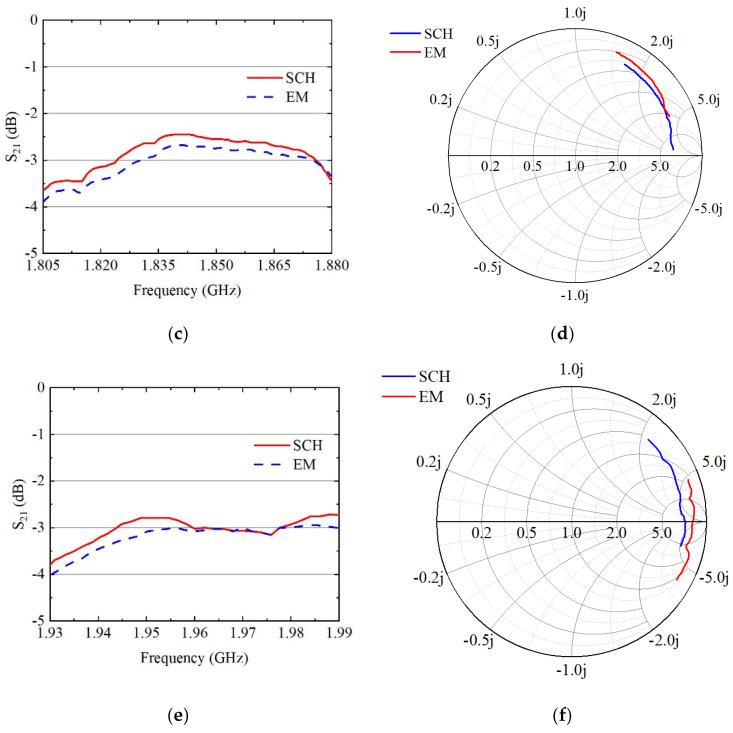
Simulation results of B0103 CA B25: (**a**) insertion loss in the B1 band; (**b**) CA impedance of B25 in the B1 band; (**c**) insertion loss in the B3 band; (**d**) CA impedance of B25 in the B3 band; (**e**) insertion loss in B25 band; (**f**) CA impedance of B0103 in the B25 band.

The results in Figure 11 indicate that there will be some deviation from the ideal scenario in the actual layout, with a more pronounced impact observed in the case of CA. To better illustrate this phenomenon, let us consider the B0103 CA B25 module as an example. An HFSS 3D EM model will be simulated based on the PCB file generated from the module schematic. The simulation results will be saved as an Snp file, which will then be imported into ADS and connected to the circuit according to the module’s schematic. Figure 12 depicts the circuit diagram, encompassing switch models, PCB models, filter models, and their corresponding matching networks. The Rx end matching employs ideal inductors and capacitors within ADS. Subsequently, the circuit illustrated in Figure 12 undergoes simulation and analysis to optimize RF circuit performance. This involves fine-tuning each parameter, particularly the matching LC values, and adjusting parameters using ‘Optim’ and ‘Goal’ settings in ADS. The optimized outcomes are presented in Figure 13.

The CA impedance of EM simulation results differs from that in the circuit schematic simulation, which affects the performance of each filter in the CA working state. From Figure 13a,c,e, it is evident that the insertion loss of each frequency band deteriorates to some extent in the case of B0103 CA B25, with a maximum deviation of approximately 0.4 dB observed between the worst-case frequency points. On the other hand, Figure 13b,d,f depict the corresponding CA impedance characteristics which reveal a reduction in reflection coefficient value γ, and the positions of the frequency points on the curve are more dispersed compared to the original circuit impedance on the Smith chart during EM simulation analysis. This contrasts with the concentrated CA impedance observed in schematic simulation results. In light of these findings, our focus lies on minimizing this effect as much as possible while striving for close alignment between EM simulation outcomes and circuit simulation results.

## 5. RF Front-End Diversity Reception Module

**Figure 14:**
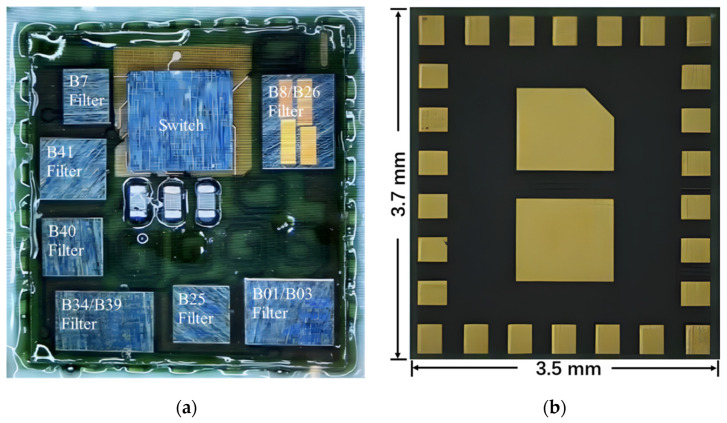
Photograph of the proposed RF diversity receiver module. (**a**) Top view. (**b**) Bottom view.

To fully validate the proposed architecture of the RF front-end diversity reception module in this paper, after conducting CA matching research and optimizing RF circuit performance, the design of the SiP substrate and prototype production of this module were carried out. All frequency bands of the filters require external matching. The module includes only the antenna-end matching of the filter. The picture of the final product after processing and manufacturing has external dimensions of 3.7 mm × 3.5 mm, meeting the demand for miniaturization of RF modules. Figure 14 showcases the photograph of the proposed RF diversity receiver module, depicting (Figure 14a) the top view and (Figure 14b) the bottom view, providing a detailed representation of the module’s physical dimensions and intricate design.

**Figure 15:**
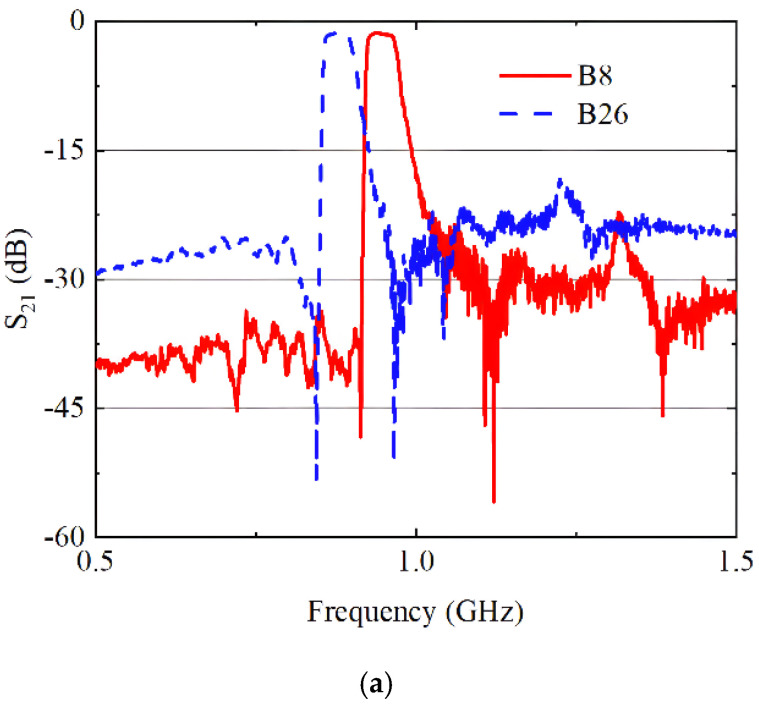

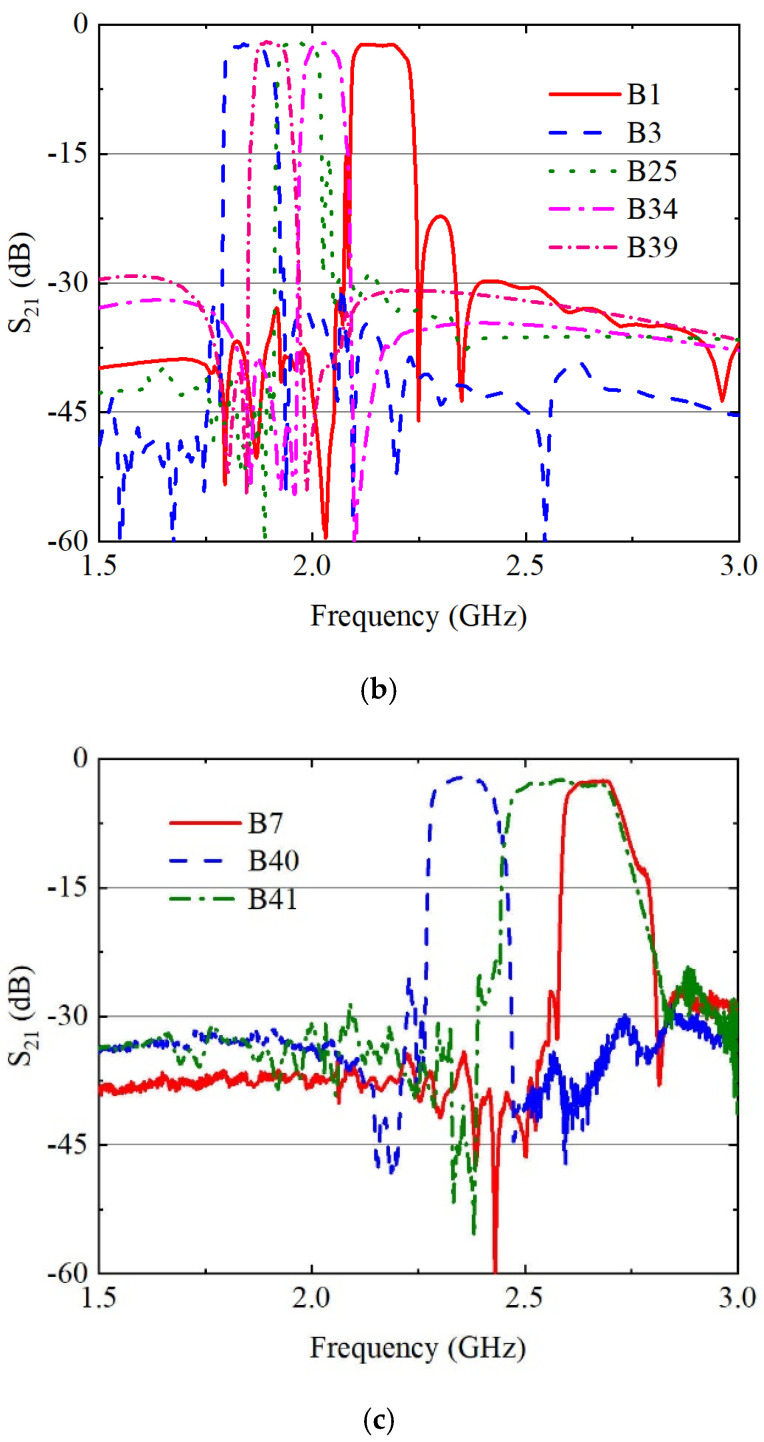
Measured insertion loss for the proposed RF diversity receiving module: (**a**) LB; (**b**) MB; (**c**) HB.

The RF front-end diversity receiving module was measured using a vector network analyzer and testing fixture, and the measured S_21_ curves are shown in Figure 15. Within its operating frequency range, this RF front-end module achieves an insertion loss of less than 5 dB, while providing more than 25 dB suppression for out-of-band signals at mid-to-high frequencies. Compared to traditional integrated RF front-end modules, this module has a smaller footprint, richer functionality, and stronger stability.

The Voltage Standing Wave Ratio (VSWR) measures the ratio of the maximum voltage to the minimum voltage along a transmission line in RF systems, reflecting the efficiency of RF power transfer from the source to the load. The operating frequency bands and VSWR of the RF diversity reception module are presented in Table 2. In Table 2, a VSWR value of less than 1.9 indicates that more than 90% of the signal is received.

## 6. Conclusions

This paper proposes a novel chip architecture that integrates SOI switches and SAW filters on SiP substrate design, aiming to achieve high integration and effective reduction in energy consumption. The trial production of the RF front-end diversity reception prototype module confirms the integration effectiveness and application feasibility of this approach, ensuring its high reliability for adoption in miniaturized communication devices. The dimensions of the diversity receiving module designed are 3.7 mm × 3.5 mm, and the working frequency bands include B26, B8, B1, B3, B25, B34, B39, B40, B41, and B7, and the working conditions of eight kinds of CA. With better insertion loss and CA performance, it can meet the operating requirements and conditions of RF diversity receiving modules in current communication systems.

## Figures and Tables

**Table 1:** Implementation of diversity receiving module.

	Band	Frequency Band (MHz)	CA Case
LB	B26	859–894	
	B8	925–960	
MHB	B3	1805–1880	B40, B41, B7, B25
	B1	2110–2200	
	B25	1930–1995	B0103, B7, B40, B41
	B34	1880–1920	B41
	B39	2010–2025	
	B40	2300–2400	B7, B25, B41
	B41	2496–2690	B0103, B3439, B40
	B7	2620–2690	B0103, B25, B40

**Table 2:** VSWR of the proposed diversity receiving module.

	Band	VSWR
LB	B8	1.81
	B26	1.86
MB	B1	1.61
	B3	1.54
	B25	1.83
	B34	1.19
	B39	1.42
HB	B7	1.34
	B40	1.47
	B41	1.88

## Data Availability

The data supporting this research article are available upon request to the corresponding author.

## References

[1] Dash L., Koithyar A. Performance investigation of diversity techniques for 5G communication scenario. Proceedings of the 3rd International Conference on Advances in Physical Sciences and Materials (ICAPSM 2022).

[2] Florinel B., Hardik M., Yun C., Jun L., Serge D., Sabah K. 5G RF Front End Module Architectures for Mobile Applications. Proceedings of the 49th European Microwave Conference (EuMC 2019).

[3] Lu P., Liu Z. Highly Integrated Multi-Channel RF Front-End Module. Proceedings of the IEEE MTT-S International Wireless Symposium (IWS 2023).

[4] Guo M., Lei X., Xiong H., Chang T., Chen Y., Lv Y., Yuan Z., He Y., Zhao Z., Jing L. A 5–6 GHz CMOS RF Front-End Module for FTTR Application. Proceedings of the IEEE MTT-S International Wireless Symposium (IWS 2023).

[5] Liao T., Lai W., Shih H.-C., Chen D., Tarng D., Hung C. Mechanical reliability analysis of dual side molding SiP module. Proceedings of the International Conference on Electronics Packaging (ICEP 2021).

[6] Lu X., Zhou S., Wei B., Zhou L. (2023). Three-dimensional SIP design of the four-channel RF transceiver based on silicon and ALN for X-band radar applications. IEEE Trans. Compon. Packag. Manuf. Technol..

[7] Wang S., Yan M., Chen H., Che W., Xue Q. Design of a miniaturized RF front-end module with antenna array for 5G millimeter-wave communication. Proceedings of the IEEE International Workshop on Electromagnetics: Applications and Student Innovation Competition (iWEM 2021).

[8] Fraser M., Malladi V., Staudinger J., Staudinger J., Chang C. A wide-band RF front-end module for 5G MIMO applications. Proceedings of the IEEE Radio Frequency Integrated Circuits Symposium (RFIC 2020).

[9] Kim Y., Han J., Lee J., Jin T., Jang P., Dhin H., Lee J., Cho T. (2021). Power-efficient CMOS cellular RF receivers for carrier aggregation according to RF front-end configuration. IEEE Trans. Microw. Theory Tech..

[10] Liu T., Du B., Zhong H., Wang H. A carrier aggregation pentaplexer with an optimized phase shifter network in BAW technologies. Proceedings of the IEEE MTT-S International Microwave Workshop Series on Advanced Materials and Processes for RF and THz Applications (IMWS-AMP 2022).

[11] Yu H., Peng J., Wang X., Tan Y., Wen P., Tao Y., Deng L., Jiang C. A gold bump flip-chip interconnection structure for face-up MMIC assembly in RF front-end modules. Proceedings of the IEEE MTT-S International Microwave Workshop Series on Advanced Materials and Processes for RF and THz Applications (IMWS-AMP 2023).

[12] Seo H., Zhou J. Periodically switched acoustic-filtering RF front-ends using commutated-LC circuits for wireless receivers. Proceedings of the IEEE 22nd Annual Wireless and Microwave Technology Conference (WAMICON 2022).

[13] Seo H., Zhou J. (2021). A passive-mixer-first acoustic-filtering superheterodyne RF front-end. IEEE J. Solid-State Circuits.

[14] Pirro M., Cassella C., Michetti G., Rinaldi M. Low loss non-reciprocal filter for miniaturized RF-front-end platforms. Proceedings of the IEEE International Frequency Control Symposium and European Frequency and Time Forum (EFTF/IFC 2019).

[15] Li B., Wu Z., Deng J., Dai D., Li B., Su Q., Peng Y. Integrated design of power amplifier and saw filters for reconfigurable RF front-end. Proceedings of the 8th International Symposium on Next Generation Electronic (ISNE 2019).

[16] Campanella H., Qian Y., Romero C.O., Wong J., Giner J., Kumar R. (2021). Monolithic multiband MEMS RF front-end module for 5G mobile. Microelectromech. Syst..

[17] Raskin J. (2022). Fully depleted SOI technology for millimeter-wave integrated circuits. IEEE J. Electron Devices Soc..

[18] Fan J., Qi B., Li X., Xu J., Zhang W., Peng Y. (2019). Design and Implementation of a Carrier Modulation MOSFET Driver Circuit. High Volt. Eng..

[19] Hussain I., Woo D.-K. (2022). Inductance Calculation of Single-Layer Planar Spiral Coil. Electronics.

